# Cohesin and chromosome segregation

**DOI:** 10.1016/j.cub.2018.05.019

**Published:** 2018-06-18

**Authors:** Vasso Makrantoni, Adele L. Marston

**Affiliations:** The Wellcome Centre for Cell Biology, Institute of Cell Biology, School of Biological Sciences, Michael Swann Building, Max Born Crescent, Edinburgh, EH9 3BF, UK

## Abstract

Cohesin is a ring-shaped protein complex that organises the genome, enabling its condensation, expression, repair and transmission. Cohesin is best known for its role in chromosome segregation, where it provides the cohesion that is established between the two newly duplicated sister chromatids during S phase. This cohesion enables the proper attachment of sister chromatids to microtubules of the spindle by resisting their opposing pulling forces. Once all chromosomes are correctly attached, cohesin is abruptly destroyed, triggering the equal segregation of sister chromatids to opposite poles in anaphase. Here we summarise the molecular functions and regulation of cohesin that underlie its central role in chromosome segregation during mitosis.

## Teaser

Cohesin is a ring-shaped protein complex that organises the genome, enabling its condensation, expression, repair and transmission. In this Primer, Makrantoni and Marston summarise the molecular functions and regulation of cohesin that underlie its central role in chromosome segregation during mitosis.

## Main Text

### Architecture of cohesin

**Figure 1:**
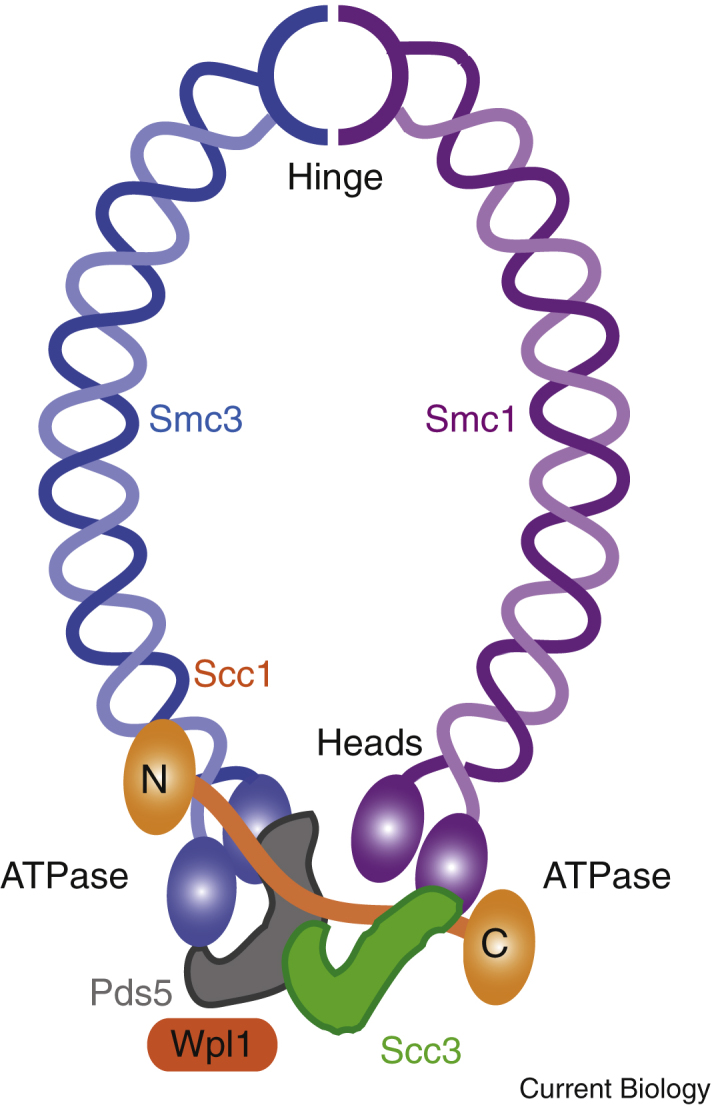
Cohesin structure in yeast. Two SMC cohesin subunits are linked tail-to-tail at the hinge domain and also at their ATPase heads, through the α-kleisin subunit, Scc1, to form a tripartite ring. Three additional accessory subunits — Scc3, Pds5 and Wpl1 — associate with the kleisin subunit.

Cohesin is a member of the ancient genome-organising SMC (Structural Maintenance of Chromosomes) family of protein complexes, present from bacteria to humans. It comprises two SMC proteins, Smc1 and Smc3, and a ‘kleisin’ subunit, Scc1, that together form a tri-partite ring (here, the budding yeast (*Saccharomyces cerevisiae*) names will be used throughout; see Table 1 for orthologs in other organisms). The Smc1 and Smc3 subunits are flexible, antiparallel coiled-coils that are linked at one end by a dimerization domain known as the ‘hinge’ to form a V-shaped structure that can open or close by virtue of this hinge domain. At the apices of the V, the SMC amino and carboxyl termini contain ATP Binding Cassette-family ATPase ‘head’ domains (Figure 1). Dimerization of the Smc1 and Smc3 heads is dependent on sandwiching two ATP molecules between them. Scc1 helps hold the heads together because its amino-terminal domain complexes with the coiled-coil proximal to the Smc3 head, and its carboxy-terminal domain interacts with the Smc1 head. Three accessory subunits, Scc3, Pds5 and Wpl1, associate with the kleisin subunit and regulate both cohesin association and dissociation with chromatin. The cohesin loader, Scc2–Scc4, also interacts with the cohesin ring to promote its association with chromatin.

**Table 1:** Cohesin subunits and regulators in different species.

Function	*S. cerevisiae*	*S. pombe*	*D. melanogaster*	*H. sapiens*
Core cohesin subunit	Smc1 Smc3 Mcd1/Scc1, ***Rec8***	Psm1 Psm3 Rad21, ***Rec8***	Smc1 Smc3 Rad21, ***C(2)M***	Smc1α, ***Smc1***b Smc3 Rad21, ***Rad21L, Rec8***
Cohesin associated	Scc3 Pds5 Rad61/Wpl1	Psc3, ***Rec11*** Pds5 Wpl1	SA Pds5 Wapl Dalmatian∗	SA1, SA2, ***STAG3*** Pds5a, Pds5b Wapl Sororin
Cohesin loading	Scc2 Scc4	Mis4 Ssl3	Nipped-B Scc4	Nipbl Mau2
Cohesin acetyltransferase	Eco1	Eso1	Deco, San	Esco1, Esco2
Cohesin deacetylase	Hos1			HDAC8

Protein names of mitosis- (plain text) or meiosis-specific (bold italic text) core and associated cohesin subunits and the proteins that regulate the association, establishment or release of cohesin in yeast (*S. cerevisiae* and *S. pombe*), flies (*D. melanogaster*) and humans (*H. sapiens*).

∗ Hybrid of sororin and shugoshin.

**Figure 2:**
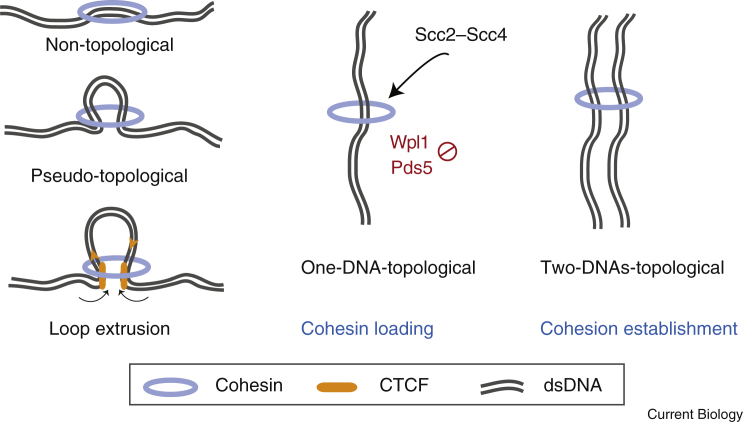
Proposed functional modes of cohesin interaction with DNA. Simple model of ‘non/pseudo-topological’ cohesin interactions that mainly drive loop extrusion, the ‘one-DNA-topological’ configuration, which is thought to be the product of cohesin loading, and the ‘two-DNAs-topological’ form, which is likely the final cohesive configuration.

The provocative shape of cohesin led to the proposal of the ‘ring model’ whereby the two sister chromatids are topologically entrapped within a single cohesin ring. Artificial cross-linking of cohesin’s three interfaces prevented its release from circular minichromosomes after denaturation, providing compelling evidence for this topological mode of interaction. The ring model has provided a useful framework to understand the molecular basis of cohesion establishment and destruction. Here we present the current state of knowledge in terms of the simplest form of the ring model, in which three functional modes of interaction can be envisaged (Figure 2): the non/pseudo-topological configuration, the one-DNA-topological configuration, and the two-DNAs-topological configuration. Non/pseudo-topological cohesin interactions drive loop extrusion, which directs chromosomal organization in interphase, but may not be directly relevant for sister chromatid cohesion, except potentially as an intermediate in the loading reaction. The one-DNA-topological configuration is thought to be the relevant product of cohesin loading that provides the precursor for establishment of sister chromatid cohesion. Finally, the two-DNAs-topological form is likely to be the final cohesive configuration. Accordingly, opening or breaking the ring causes loss of sister chromatid cohesion. Although this single-ring model prevails, and is attractive in its simplicity, intra-allelic complementation experiments have raised the possibility that multiple cohesin rings collaborate in cohesion generation so more elaborate cohesin–DNA configurations may also be functionally important.

### Cohesin loading

**Figure 3:**
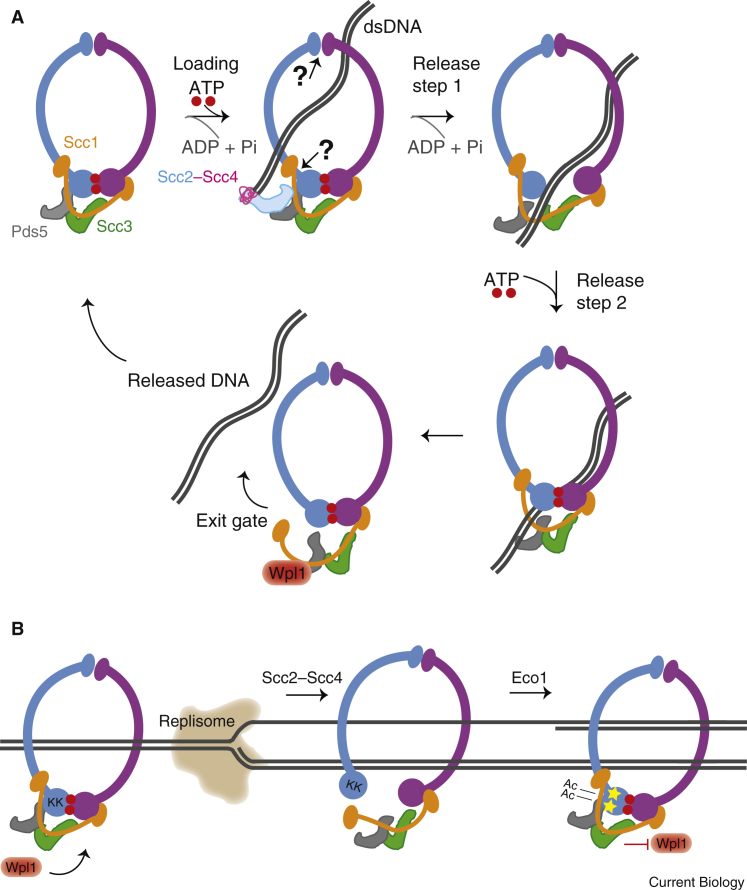
Cohesin dynamics throughout the yeast cell cycle. (A) Cohesin loading on chromatin is mediated by the loading complex Scc2–Scc4 and requires ATP binding at the SMC heads. ATP hydrolysis and re-binding ensures chromosome entrapment through a speculative ‘entry gate’. The turnover of cohesin on chromosomes is facilitated by Wpl1 and Pds5 and DNA is released through the ‘exit gate’ located at the Smc3–Scc1 interface. (B) In S phase, cohesion establishment is linked to DNA replication and requires first, that the cohesin–DNA configuration is such that both sister DNA molecules are entrapped within the ring, and second, that the ring remains shut, preventing the release of the DNA molecules. The latter step requires Eco1-dependent acetylation of two lysine residues at the Smc3 head domain, making cohesin refractory to Wpl1.

To provide cohesion, cohesin must be loaded onto chromatin before DNA replication in S phase. The most likely outcome of cohesin loading is the topological interaction of cohesin rings with individual (that is, unreplicated) double-stranded DNA molecules, though non-topological cohesin–chromatin interactions may serve as important intermediates. Loading depends on an essential conserved cohesin-loader complex termed Scc2–Scc4 in budding yeast (Nipbl–Mau2 in mammals, Table 1) and hydrolysis of ATP by the SMC heads. The Scc2 subunit confers the loading activity and is sufficient for the topological association of cohesin with DNA *in vitro*. Although it does not participate directly in the loading reaction, the other subunit of the cohesin loader, Scc4, stabilizes Scc2 *in vivo* and is also important in targeting the cohesin-loader complex to chromatin (see below). Cohesin loading occurs in two steps (Figure 3A). First, Scc2 binds — via its hook-like domain — to the amino terminus of Scc1 in the assembled cohesin ring, with the ATP-bound heads engaged (though other contact sites on cohesin may also be important). Second, Scc2 promotes cohesin’s ATPase activity, which is expected to drive the heads apart to trigger a conformational change, resulting in opening of the cohesin ring to allow DNA entry. However, the identity of the subunit interface — known as the ‘gate’ — that opens to allow DNA entry is debated. One view is that DNA enters through the Smc3–Scc1 interface, which is widely accepted to be the DNA-exit gate and could therefore involve a near reversal of the two-step mechanism of cohesin release (see below). In support of this idea, the requirements for DNA entry and exit are similar in biochemical experiments and the binding of Scc2 to Scc1, close to the ATPase heads, could easily be envisaged to induce an ATP-dependent conformational change at the Smc3–Scc1 gate. An alternative proposal is that the cohesin hinge is the site of DNA entry. Support for this idea came from the demonstration that artificial tethering of the Smc1 and Smc3 hinge domains prevented cohesin loading, whereas closure of the Smc1–Scc1 or Smc3–Scc1 interfaces did not, which is difficult to reconcile with the idea that DNA enters through an Smc3–Scc1 entry gate.

Whether it opens or not, the hinge is clearly important in the loading reaction. First, the fission yeast (*Schizosaccharomyces pombe*) hinge domain forms a supramolecular complex with fission yeast Scc3 and the cohesin loader, leading to suggestions that the cohesin ring can fold back on itself. Second, specific mutations in the hinge domain prevent DNA entrapment, but not ATP hydrolysis or cohesin translocation along DNA. Interestingly, these hinge mutants retain the ability to condense chromatin, despite not being able to entrap DNA or provide cohesion. This provides evidence that non-topological interactions of cohesin with chromatin exist *in vivo* and are sufficient to drive genome organization. Cohesion, however, relies on DNA entrapment.

### Cohesin loading sites

In budding yeast, cohesin-loading sites on chromosomes are selected via two modes: targeted and general. The targeted mode, which is best understood, occurs at the ∼125 bp centromere and is dependent on a conserved surface patch on the Scc4 subunit of the cohesin loader, the Ctf19 inner kinetochore sub-complex, and the Dbf4-dependent kinase (DDK). Targeting of cohesin loading occurs in two steps. First, DDK binds Ctf3, a subunit of the Ctf19 kinetochore sub-complex, and phosphorylates the amino terminus of the Ctf19 subunit. Second, the conserved Scc4 patch docks onto the phosphorylated Ctf19 amino terminus. This kinetochore-driven mechanism of cohesin loading enriches cohesin throughout the ∼20 kb surrounding pericentromere and is thought to facilitate proper kinetochore–microtubule interactions to promote accurate chromosome segregation. How kinetochore-loaded cohesin spreads into the pericentromere is not well understood, but ATP-dependent translocation of cohesin along chromatin, which has been observed by *in vitro* single molecule experiments, is an attractive possibility. In contrast to cohesin, the Scc2–Scc4 loader does not move away from loading sites, and cohesin-bound Scc2 is exchanged for Pds5, which competes for the same binding site on cohesin. This not only frees up Scc2 to participate in further loading reactions but also renders chromosome-bound cohesin susceptible to both positive and negative regulation via Pds5.

In addition to targeted cohesin loading at the centromere, general, untargeted cohesin loading occurs genome-wide in budding yeast, but is much less understood and independent of the targeting function of Scc4. Promoters of highly transcribed genes, such as tRNAs and ribosomal genes, have emerged as potential favorable cohesin-loading sites. The nucleosome remodeler RSC, which generates nucleosome-free regions at promoters, has been implicated in recruitment of the Scc2–Scc4 loader. Since the majority of *in vivo* studies of cohesin loading have concentrated on centromeric mini-chromosomes or analysis of the endogenous pericentromeric region, a key goal will be to understand whether the same principles apply genome wide.

Some aspects of the targeted cohesin-loading mechanism at the budding yeast centromere are likely to be conserved in other systems. A role for DDK in targeting cohesin loading is a common theme, though the relevant substrates and the important interactions have not yet been clearly defined. Association of cohesin with chromatin in *Xenopus laevis* egg extracts requires DDK and the conserved patch on Scc4. In human cells, DDK and phosphorylated replicative helicase, Mcm2-7, are important for cohesin association with chromosomes, though this may be more important in cohesion establishment — that is, the conversion from the one-DNA-topological form to the two-DNAs-topological form of cohesin (see below) — rather than the initial loading of cohesin onto chromosomes. Translocation of cohesin away from its initial loading sites has also been observed in mammalian cells, and is likely to be important in structuring the genome. Scc2 and cohesin co-localize at core promoter and enhancer sites with the transcriptional co-activator mediator, and probably represent loading sites, reminiscent of the situation in yeast. Cohesin, but not Scc2, additionally co-localizes with the transcription factor CTCF, where it tethers chromatin fibres at the base of a chromatin loop to facilitate long-range interactions. A loop extrusion mechanism, which has recently been visualized for the related condensin complex, is likely responsible. Starting with a pseudo-topological cohesin interaction at the mediator-bound core promotor/enhancer, Scc2-stimulated ATPase activity drives loop elongation until cohesin encounters inward-pointing CTCF, which acts as a boundary to further extrusion and forms the base of the loop (Figure 2). Wapl counteracts loop formation by promoting cohesin turnover, while Pds5 is important for boundary function together with CTCF, perhaps by preventing cohesin turnover. Although this loop extrusion mechanism can explain how chromosome organisation is achieved in interphase, its relevance for the cohesin that participates in cohesion is not yet clear.

### Establishment of cohesion

Loaded cohesin is unstable on chromosomes and this instability is promoted by the accessory subunit Wpl1, which together with Pds5 counteracts the loading activity of Scc2–Scc4, resulting in dynamic turnover of cohesin on chromatin prior to S phase. The Wpl1–Pds5 complex promotes DNA release through the exit gate at the Smc3–Scc1 interface. The releasing reaction might be triggered because DNA inside the cohesin ring stimulates the ATPase activity of the SMC heads and drives them apart. This ‘heads-disengaged’ form of cohesin has been suggested to enable Wpl1–Pds5 binding to Scc3 and hold Scc1 in a rigid scaffold, favoring the opening of the Smc3–Scc1 interface and DNA release.

During S phase, two things need to happen to produce stable cohesion, and both of these are coupled to DNA replication (Figure 3B). First, the cohesin–DNA configuration generated during cohesin loading, presumably the one-DNA-topological form, must be converted to the two-DNAs-topological form so that both sister DNA molecules are entrapped within the ring. Second, immediately thereafter, the ring must be locked shut and made resistant to Wpl1–Pds5 so that the two DNA molecules cannot escape. A recent elegant study provided a molecular explanation of how a one-DNA-topological cohesin ring might entrap the second nascent DNA molecule at the replication fork. Cohesin is proposed to again open in an ATP- and Scc2-dependent manner — similar to the initial loading step, except that single-stranded DNA is the obligate template for capture of the second strand. Concomitant lagging-strand DNA synthesis could be envisaged to generate two DNA duplexes entrapped within a single ring. Ring locking would also be expected to occur simultaneously with second-end capture. In yeast and mammals, ring locking depends on acetylation of two highly conserved lysine residues (K112 and K113 in budding yeast) in the Smc3 head. In budding yeast, Eco1-dependent acetylation appears to be coupled to replication-fork progression and is alone sufficient to counteract Wpl1–Pds5. The acetylated lysine residues are close to the ATPase site and are thought to prevent DNA-stimulated ATP hydrolysis, thus ensuring that the heads remain engaged and the ring tightly closed and resistant to Wpl1–Pds5. In vertebrates, however, there are two acetyltransferases, Esco1 and Esco2 (Table 1), and substantial Smc3 acetylation exists already prior to S phase so that — although acetylation is required for ring-locking — it does not appear to be the critical DNA replication-coupled step. Instead, an unknown process coupled to DNA replication enables the acetylation-dependent association of a further protein, sororin, which is not found in yeast. Sororin is essential to counteract the releasing activity of Wapl in vertebrates by competing for binding to Pds5. Since the majority of Smc3 acetylation appears dependent on Esco1, it remains possible that the critical replication-coupled step in recruiting sororin is the *de novo* acetylation of a minor pool of previously unacetylated cohesin at the replication fork by the Esco2 acetyltransferase, which is active only in S phase in *Xenopus* egg extracts. Conversely, Esco1-dependent acetylation might be important in stabilizing topological interactions of cohesin during interphase.

Cohesion can be established in the absence of Eco1 in budding yeast so long as Wpl1 is also absent, though it is clearly less robust than normal. Following the model above, this could be explained by second-strand DNA capture to generate the two-DNAs-topological cohesin rings which, due to lack of acetylation, would not be protected from DNA-stimulated ATP hydrolysis. Consequently, disengagement of the Smc3 heads could occur but, in the absence of Wpl1, the open conformation would not be stabilized, and the Smc3–Scc1 interface would preferentially remain closed.

### Cohesin release

Following S phase, a minor fraction of cohesin will be in the two-DNAs-topological form, acetylated and, in mammals, bound to sororin. This Wpl1-resistant pool of cohesin provides sister chromatid cohesion. The remaining major pool of cohesin, which could include all forms of topological and non-topological interactions, is not cohesive and is therefore susceptible to destabilization by Wpl1. In mammalian cells, but not yeast, the Wapl-sensitive pool is removed from chromosomes in mitotic prophase, leaving only the acetylated, sororin-bound pool at metaphase. This so-called ‘prophase pathway’ of cohesin removal gives mitotic chromosomes their typical X-shaped structure and probably helps to organise chromosomes for their segregation. The prophase pathway relies on the mitotic kinases CDK1 and PLK1, which phosphorylate sororin and Scc3, respectively, to trigger Wapl-dependent cohesin removal. Phosphorylation of sororin disrupts its interaction with Pds5, providing access to Wapl and cohesin release. In this way, mitotic kinases trigger cohesin removal on chromosome arms. However, at centromeres, a complex of shugoshin (Sgo1) and protein phosphatase 2A counteracts this prophase pathway of cohesin release, to ensure that sister chromatids remain cohesed until properly aligned on the spindle at metaphase. In addition to phosphorylating sororin on chromosome arms, CDK1 also phosphorylates Sgo1, allowing it to bind the Scc1 and Scc3 cohesin subunits. Protein phosphatase 2A dephosphorylates sororin thereby maintaining its association with Pds5 and rendering it inaccessible to Wapl. In this way, centromeric cohesion is protected from the prophase pathway of cohesin removal, and sister chromatids remain cohered and ready for segregation.

### Cohesion destruction

The ultimate goal of cohesion is the equal segregation of the sister chromatids to opposite poles. Individual, acetylated cohesin rings in the two-DNAs-topologically associated form need to resist the pulling forces of the spindle as sister chromatids attach to microtubules from opposite poles. Once this has occurred, cohesin must be destroyed so that the sister chromatids can move to opposite poles. Molecularly, this means opening the cohesin ring, but in this case it is *not* dissociation of its subunits at one of its three gates that is responsible. Instead, all chromosomal cohesin is abruptly cut open by a protease, releasing the sister chromatids and resulting in their sharp and coordinated movement to opposite poles. The protease is called separase, and its target is the cohesin subunit Scc1. This process can be mimicked in yeast, *Drosophila* and mammalian cells by engineering a cleavage site for the ectopically expressed TEV protease into Scc1. This demonstrates that cohesin cleavage is the trigger for chromosome segregation in a wide range of species, whether the prophase pathway of cohesin removal exists or not. Accordingly, separase activity is tightly controlled; separase is inactive before all chromosomes are properly attached to the spindle because it is bound to an inhibitory chaperone called securin and, in mammalian cells, CDK1. Once chromosomes are properly bioriented the so-called ‘spindle checkpoint’ is satisfied and this permits activation of APC/C, an E3 ubiquitin ligase that polyubiquitylates both securin and CDK1-associated cyclin B and targets them for destruction by the 26S proteasome. This liberates separase to cleave cohesin.

The final step in the cohesin cycle is the deacetylation of the cohesin subunit Smc3 upon its release from chromosomes by the Hos1 deacetylase. This both allows its recycling for the next cell cycle (cohesin must be deacetylated at the K112 and K113 residues to be loaded on to chromosomes) and also promotes efficient loss of cohesion during anaphase.

### Conclusions and perspectives

Remarkable progress has been made in understanding how cohesin provides chromosome cohesion. We now have a clear framework linking molecular mechanism to cellular function with implications for SMC protein function beyond sister chromatid cohesion. Further structural, biochemical, and single molecule approaches, together with cell biology and specifically engineered mutants, promise to fill gaps in our knowledge as to the intricate workings of this fascinating molecular machine and its regulators. Mutations in cohesin regulators cause severe developmental disorders including Cornelia de Lange syndrome (Scc2/Nipbl) and Roberts syndrome (Esco2). A detailed molecular knowledge of cohesin mechanism will be essential in defining how specific disease-causing mutations impinge on function and human development.
